# Supplementary material for: Identifying a Common Functional Framework for Apathy Large-Scale Brain Network
Source: J Pers Med. 2021 Jul 19;11(7):679. doi: 10.3390/jpm11070679 (PMC8303126; doi:10.3390/jpm11070679)
Supplement: Supplementary file 1 [file jpm-11-00679-s001.zip › jpm-1258912-supplementary.pdf]

Supplementary Materials:

Table S1. fMRI seed-to-seed total results from resting-state paradigm with functional connectivity between apathetic patients and HC groups (higher connectivity between seeds have a positive value of T-score, lower connectivity have a negative value of T-score).

	**Apathetic patients > HC**		
**Seed**	**Targets**	**T-score**	**p-FDR**
Frontal Pole right	aSMG left	-4.3	0.013
	aSMG right	-4	0.013
Postcentral Gyrus left	aSMG left	4.2	0.014
central opercular left	frontal operculum right	3.5	0.043
	aParahippocampal gyrus right	-4.2	0.007
	Putamen left	3.5	0.043
Planum Polare left	aParahippocampal gyrus right	-4.3	0.007
	Planum temporale right	-3.8	0.015
	SMA right	-3.6	0.017
	Temporal pole right	-3.4	0.029
	subcallosal cortex	-3.1	0.046
	Postcentral Gyrus left	-3.1	0.046
	inferior temporal gyrus right	-3	0.048
	caudate left	3	0.048
Heschl's gyrus left	Heschl's gyrus right	-3.9	0.032
	aSTG	-3.7	0.032

Table S2. fMRI seed-to-seed total results from resting-state paradigm with functional connectivity between apathetic FTD-PD and HC groups (higher connectivity between seeds have a positive value of T-score, lower connectivity have a negative value of T-score).

	**Apathetic FTD > HC**		
**Seed**	**Targets**	**T-score**	**p-FDR**
Insular cortex left	caudate left	4.3	0.023
Postcentral gyrus right	frontal orbital cortex left	-3.8	0.041
	hippocampus left	-3.6	0.047
frontal orbital cortex left	aSTG left	3.9	0.042
Planum Polare left	Postcentral gyrus left	-3.6	0.048
	caudate left	3.5	0.048
	**Apathetic PD > HC**		
**Seed**	**Targets**	**T-score**	**p-FDR**
Frontal Pole right	aSMG left	-4	0.02
	aSMG right	-4	0.02
Temporal pole right	parietal operculum left	-4	0.04
pSTG right	planum polare right	-4.1	0.026
	central opercular right	-3.9	0.026
pMTG right	planum polare right	-3.5	0.048
	planum temporale left	-3.5	0.048
	parietal operculum left	-3.4	0.048
postcentral gyrus left	aSMG left	4.3	0.018
aParahippocampal gyrus right	central opercular left	-4.3	0.018
lingual gyrus left	accumbens left	4	0.039
Planum Polare left	planum temporale right	-3.7	0.021

	pMTG right	-4.1	0.016
	Temporal pole right	-3.6	0.021
	aParahippocampal gyrus right	-3.5	0.022
	aSTG left	-3.3	0.04
	SMA right	-3.2	0.045
	pITG right	-3.1	0.045
	hippocamus right	-3.1	0.045
Heschl's hyrus right	aSTG left	-3.7	0.041
Heschl's hyrus left	Heschl's hyrus right	-4	0.034
	pSTG right	-3.7	0.044
	pMTG right	-3.3	0.046
	planum temporale right	-3.3	0.046
	aSTG left	-3.3	0.046
	SMA left	-3.3	0.046
